# Impact of p53 status on recurrence patterns and oncologic outcomes in non-invasive high-grade endometrial cancer

**DOI:** 10.3389/pore.2026.1612543

**Published:** 2026-08-31

**Authors:** Eduardo Paulino, Thiago Branco, Guilherme Gomes de Mesquita, Andreia Cristina de Melo

**Affiliations:** Division of Clinical Research and Technological Development, Brazilian National Cancer Institute, Rio de Janeiro, Brazil

**Keywords:** Early stage, endometrial cancer, molecular analysis, p53, recurrence rate

## Abstract

**Objective:**

This retrospective study evaluated the impact of p53-abnormal (p53abn) status on recurrence patterns and outcomes among patients with non-invasive, high-grade endometrial cancer (EC) treated at the Brazilian National Cancer Institute (INCA) between 2010 and 2025.

**Methods:**

Eligible patients had high-grade histologies—including endometrioid grade 3 (ECG3), serous (USC), clear cell (CC), carcinosarcoma (CS), mixed, or undifferentiated tumors—without myometrial invasion. Clinical, surgical, and pathological characteristics, as well as recurrence and survival data, were reviewed. Immunohistochemical analysis of p53 expression was performed, and p53 status was correlated with clinical outcomes, particularly recurrence rate.

**Results:**

Fifty-nine patients met the inclusion criteria, with a mean age of 65 years. Most underwent total hysterectomy with or without bilateral salpingo-oophorectomy (59.3%); 40.7% also received lymphadenectomy or omentectomy. Histologic distribution was mainly endometrioid (39.0%) and serous (39.0%). Forty-six (78%) and 13 (22%) had p53 abnormal and normal tumors. Seven patients (11.9%) received chemotherapy and 15 (25.4%) received radiotherapy. Three patients had already disease outside uterus. Among 56 patients with FIGO stage IC disease, recurrence occurred in 16.1%, predominantly at extra-pelvic sites (33.3% abdominal, 22.2% distant). Five-year recurrence was 23.2% for p53abn tumors versus 0% for wild-type, and 12.5% of patients died—mostly within the p53abn subgroup.

**Conclusion:**

The findings suggest that even in non-invasive high-grade EC, p53 abnormalities confer higher recurrence risk and poorer outcomes, while wild-type tumors show excellent prognosis. These results support individualized treatment approaches and highlight the need for larger, prospective validation studies.

## Highlights

- Endometrial cancer restricted to endometrium is not well described in the literature. and retrospective studies show contradictory results.
- P53 abnormal tumors without myometrial invasion demonstrates high recurrence rate while p53 wild type tumors showed excellent outcomes regardless of histology.

## Introduction

Endometrial cancer (EC) is the most common gynecologic malignancy worldwide in developed countries and the second most common worldwide [1]. Although most cases are well-differentiated (G1, G2) endometrioid adenocarcinomas, which generally confer a favorable prognosis, non-endometrioid subtypes such as serous (USC) and clear cell carcinomas are associated with aggressive clinical behavior and disproportionate mortality [2]. The advent of molecular classification has provided a reproducible framework for risk stratification and treatment planning in EC. The Cancer Genome Atlas (TCGA), using next-generation sequencing, identified four molecular subtypes: *POLE* (DNA Polymerase Epsilon) ultra-mutated (with favourable prognosis), microsatellite instable hypermutated (intermediate prognosis), copy number low (non-specific molecular profile, intermediate prognosis) and copy number high tumors (characterized by p53 mutation and high somatic copy number alterations). This aberrations confer poor clinical outcomes regardless of histotype or grade [3]. Using a more pragmatic approach, the Canadian group was able to recapitulate the same molecular subgroups performing immunohistochemistry for p53 and mismatch repair proteins and sequencing only the *POLE* gene (10.1038/bjc.2015.190). Serous endometrial intraepithelial carcinoma (SEIC) is a distinct precursor or non-invasive lesion that mimics invasive serous carcinoma morphologically and molecularly, sharing *TP53* mutations and poor prognosis when associated with extrauterine disease. Although classified by WHO as potentially malignant with metastatic potential, its optimal management remains uncertain [2].

Recently, the new FIGO 2023 staging system for EC cancer gathered all high-grade histologies without myometrial invasion as stage IC [4]. The goals of the new staging system are to better define prognostic groups and create substages that indicate more appropriate surgical, radiological, and systemic therapies. The National Comprehensive Cancer Network guidelines suggest performing the surgical staging and the molecular classification for all endometrial carcinomas; nevertheless does not tailor the treatment based on these findings [5]. Although current European guidelines recommend treating all patients with p53-abnormal (p53abn, proficient mismatch repair protiens [pMMR]/POLE not mutated) EC and myometrial invasion as high risk (combining chemotherapy and radiotherapy), there is a lack of evidence for optimal management of stage IC tumors due to limited robust data to guide adjuvant therapy, and surveillance strategies, leading to variability in clinical practice [6].

Clarifying the natural history, recurrence risk, and benefits of multimodal therapy in non-invasive high-grade EC histologies is essential to refine risk stratification and align clinical management with the molecular underpinnings of the disease. This study aims to evaluate the surgical and postoperative treatment, recurrence rate and patterns, and their association with p53abn status, in patients with non-invasive EC with high grade histologies treated at the Brazilian National Cancer Institute.

## Methods

This study was approved by the Institutional Ethics Committee (approval number 26543019.5.0000.5274), all human-related procedures were performed in accordance with institutional guidelines and followed the Good Clinical Practices.

### Patient selection

Patients with EC treated at the Brazilian National Cancer Institute (INCA) between 2010 and 2025 were retrospectively evaluated. Patients included in this analysis had high-grade histologies (endometrioid grade 3 [ECG3], serous [USC], clear cell [CC], carcinosarcoma [CS], mixed and undifferentiated) with no myometrial invasion. Clinical and pathological characteristics, including age, performance status (PS), surgical and adjuvant treatment (systemic therapy or radiotherapy), as well as patterns of relapse and survival, were retrieved from the medical records. The International Federation of Gynecology and Obstetrics (FIGO) 2023 staging system for endometrial cancer was used for staging purposes.

### Immunohistochemistry analysis

All immunohistochemistry (IHC) was performed for the purposes of the present study. Analyses were performed on paraffin-embedded specimens sectioned at 4 μm and incubated at 60 °C for at least 2 h. Sections were deparaffinized in xylene and rehydrated. The slides were then placed in a steamer containing Trilogy™ solution (Cell Marque, Rocklin, CA, USA) for 30 min for antigen retrieval. After cooling to room temperature, the slides were subjected to IHC using a commercial peroxidase polymer-based kit according to the manufacturer’s instructions (Novolink Max Polymer Detection System, Leica Biosystems). Slides were incubated overnight at 4 °C with anti-p53 antibody (clone DO-7, Novocastra, Leica Biosystems) at a dilution of 1:1000. A human breast tissue sample known to be positive for the antibody was included in all runs as a positive control. For the negative control, the positive sample was processed without the primary antibody. Counterstaining was performed with Harris hematoxylin. Abnormal p53 expression was defined by one of the following patterns: strong diffuse staining in 80%–100% of tumour cell nuclei (overexpression), complete absence of staining in tumour cell nuclei in the presence of positive internal control staining (null expression), or abnormal cytoplasmic staining. Staining in 1%–80% of nuclei with variable intensity was considered wild-type p53 expression. The slides were analysed under an optical microscope by an experienced pathologist.

Unfortunately, due to financial constraints, we could not perform other biomarkers for the purposes of molecular classification.

### Definition and types of recurrence

Disease recurrence was defined as pelvic, abdominal, or distant progression. Pelvic recurrence included vaginal and local recurrences, including pelvic lymph nodes and local extension to the rectum and bladder. Recurrences outside the pelvis consisting of peritoneal carcinomatosis or omental metastasis were classified as abdominal recurrence. Distant recurrence included lung, liver, bone, and brain metastases, as well as non-pelvic or para-aortic lymph node involvement. Simultaneous pelvic and abdominal recurrence was classified as abdominal recurrence; simultaneous pelvic and distant recurrence was classified as distant recurrence; and simultaneous abdominal and distant recurrence was also classified as distant recurrence. Recurrence-free survival (RFS) was defined as the time from surgery to the date of recurrence confirmation by imaging or clinical assessment (local or distant), or death from any cause, with censoring of patients who were alive and recurrence-free. Overall survival (OS) was defined as the time from surgery to death from any cause, with censoring of patients who were alive at the date of last follow-up.

### Statistical analysis

Categorical variables were analysed with a χ2 statistic exact test. Recurrence-free survival was analysed using the Kaplan-Meier method and log-rank test. P values <0.05 were considered statistically significant. All tests were performed using the Stata software version 18.

## Results

Fifty-nine patients met the inclusion criteria and were included in this cohort. The mean age was 65 years. Surgical management varied, with most undergoing total hysterectomy with or without bilateral salpingo-oophorectomy (59.3%), while 40.7% received additional lymphadenectomy with or without omentectomy. Nearly all patients presented with stage I disease (94.9%); nonetheless three patients already had disease outside the uterus, despite the absence of myoinvasive disease in the uterine specimen. Twenty-eight (47.5%) patients had disease in a polyp and 31 (52.5%) limited to endometrium. Histologically, tumors were distributed between ECG3 (39.0%) and USC (39.0%), with fewer cases of CC, CS, or mixed subtypes. Adjuvant therapy was not uniformly applied. Only 11.9% of patients received chemotherapy (followed or not by external beam radiotherapy or brachytherapy) and 25.4% with external beam radiotherapy and/or brachytherapy. Treatment patterns were similar across p53abn and wild-type groups. Table 1 describes the cohort main characteristics.

**TABLE 1:** Main characteristics of the cohort.

Characteristics	Summary
N	59
Mean age - years (SD)	64.7 (11.6)
Performance status (ECOG)	
0/1	56 (94.9%)
2/3	3 (5.1%)
Surgical approach	
TH +/- BSO	35 (59.3%)
TH + BSO + LN +/- omentectomy	24 (40.7%)
Histology	
Endometrioid	23 (39.0%)
Serous	23 (39.0%)
Clear cell	4 (6.8%)
Carcinosarcoma	4 (6.8%)
Mixed	5 (8.5%)
FIGO stage (FIGO 2023)	
I	56 (94.9%)
II/III	3 (5.1%)
Confined polyp or endometrium	
Polyp	28 (47.5%)
Endometrium	31 (52.5%)
p53 status	
Wild type	13 (22.0%)
Abnormal	46 (78.0%)
Adjuvant treatment	
None	37 (62.7%)
Chemo +/- EBRT/BT	7 (11.9%)
EBRT and/or BT	15 (25.4%)
Progression	
No	48 (81.4%)
Yes	11 (18.6%)
Site progression	
Pelvic	5 (45.5%)
Abdominal	4 (36.4%)
Distant	2 (18.2%)
Death	
No	49 (83.1%)
Yes	10 (16.9%)

TH, total hysterectomy; BSO, bilateral salpingo-oophorectomy; LN, lymphadenectomy; SD, standard deviation, Chemo, chemotherapy; EBRT, external beam radiotherapy; BT, brachytherapy.

**FIGURE 1:**
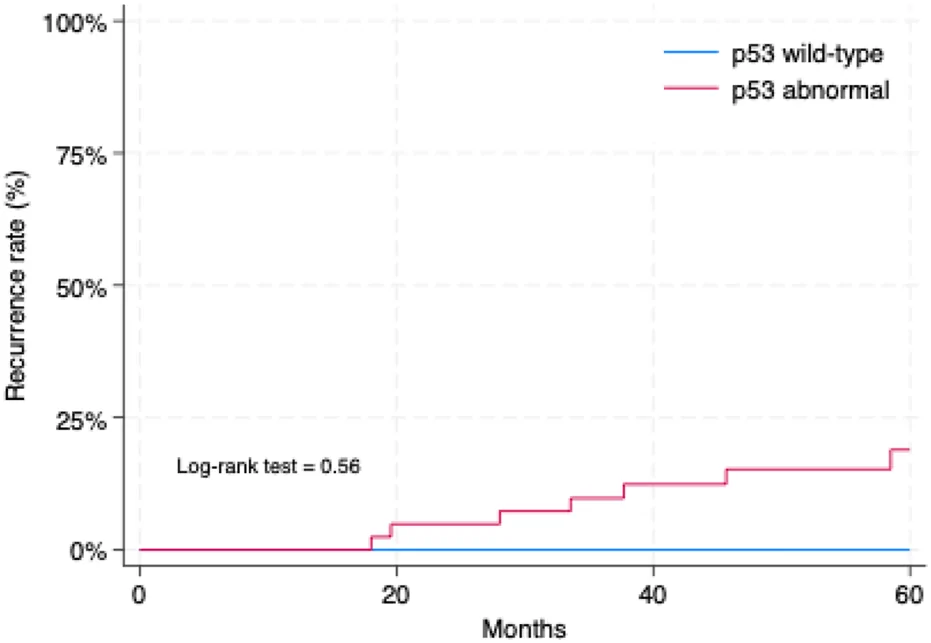
Recurrence rate in patients with FIGO IC.

**FIGURE 2:**
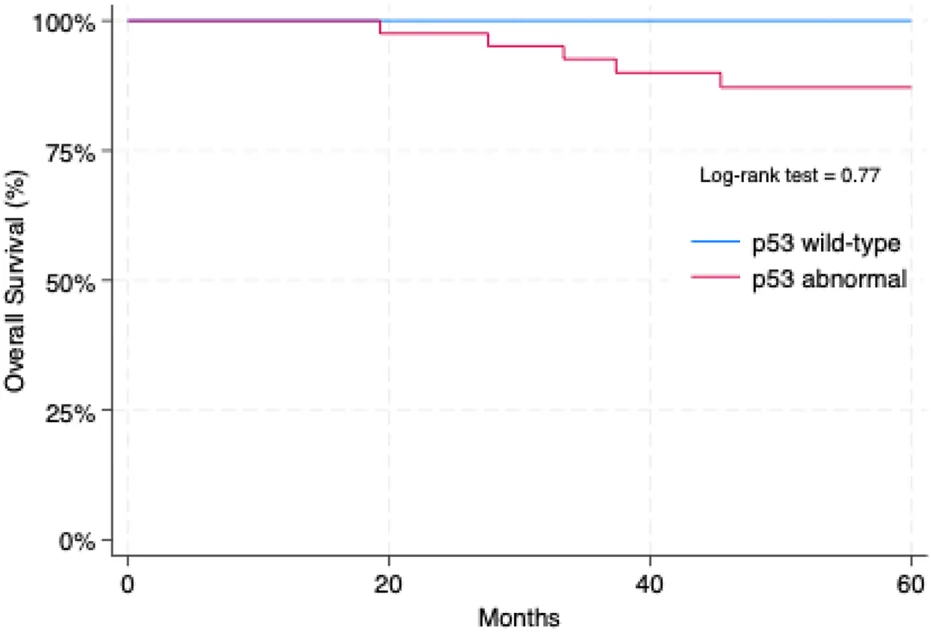
Overall survival in patients with FIGO stage IC.

Restricting the analysis only in patients with FIGO stage IC (56 patients), disease recurrence occurred in 16.1% (1 ECG3, 5 USC, 2 CC, and 1 mixed histologies) and the recurrences were predominantly outside the pelvis (33.3% in the abdomen and 22.2% at distant sites). The median follow-up, estimated using the reverse Kaplan-Meier method, was 65.4 months (95% CI, 59.2–68.7 months). Kaplan–Meier curves demonstrated differences in RFS (Figure 1) and OS (Figure 2) according to p53 status, although not statistically significant (p = 0.56 and 0.77). Patients with wild type p53 had excellent outcomes, with no patient recurring at 5 years. In contrast, the p53abn subgroup experienced an estimated 5-year recurrence rate of approximately 23.2%. In this subgroup, 5 patients received no treatment, 2 received chemotherapy with or without radiotherapy/brachytherapy and 2 received radiotherapy/brachytherapy alone. At the last follow-up, seven patients (12.5%) had died, including six with p53abn and one with wild-type disease. Table 2 summarizes the patients characteristics with FIGO 2023 IC according to recurrence and Table 3 according to p53 status.

**TABLE 2:** Patients characteristics with FIGO 2023 IC according to recurrence.

Characteristics	Disease recurrence			
​	No	Yes	Total	p-value
N	47 (83.9%)	9 (16.1%)	56 (100.0%)	​
Mean age - years (SD)	63.5 (11.7)	71.9 (10.4)	64.9 (11.8)	0.051
Performance status (ECOG)				
0/1	45 (95.7%)	8 (88.9%)	53 (94.6%)	0.403
2/3	2 (4.3%)	1 (11.1%)	3 (5.4%)	​
Surgical approach				
TH +/- BSO	27 (57.4%)	5 (55.6%)	32 (57.1%)	0.916
TH + BSO + LN +/- omentectomy	20 (42.6%)	4 (44.4%)	24 (42.9%)	​
Histology				
Endometrioid	22 (46.8%)	1 (11.1%)	23 (41.1%)	0.101
Serous	15 (31.9%)	5 (55.6%)	20 (35.7%)	​
Clear cell	2 (4.3%)	2 (22.2%)	4 (7.1%)	​
Carcinosarcoma	4 (8.5%)	0 (0.0%)	4 (7.1%)	​
Mixed	4 (8.5%)	1 (11.1%)	5 (8.9%)	​
Confined polyp or endometrium				
Polyp	21 (44.7%)	5 (55.6%)	26 (46.4%)	0.549
Endometrium	26 (55.3%)	4 (44.4%)	30 (53.6%)	​
p53 status				
Wild type	13 (27.7%)	0 (0.0%)	13 (23.2%)	0.072
Abnormal	34 (72.3%)	9 (100.0%)	43 (76.8%)	​
Adjuvant treatment				
None	31 (66.0%)	5 (55.6%)	36 (64.3%)	0.476
Chemo +/- EBRT/BT	4 (8.5%)	2 (22.2%)	6 (10.7%)	​
EBRT and/or BT	12 (25.5%)	2 (22.2%)	14 (25.0%)	​
Site progression				
Pelvic	0 (0.0%)	4 (44.4%)	4 (44.4%)	NA
Abdominal	0 (0.0%)	3 (33.3%)	3 (33.3%)	​
Distant	0 (0.0%)	2 (22.2%)	2 (22.2%)	​
Death				
No	46 (97.9%)	3 (33.3%)	49 (87.5%)	<0.001
Yes	1 (2.1%)	6 (66.7%)	7 (12.5%)	​

TH, total hysterectomy; BSO, bilateral salpingo-oophorectomy; LN, lymphadenectomy; SD, standard deviation, Chemo, chemotherapy; EBRT, external beam radiotherapy; BT, brachytherapy.

**TABLE 3:** Patients characteristics with FIGO 2023 IC according to p53 status.

Characteristics	p53 status			
​	Wild-type	Abnormal	Total	Test
N	13 (23.2%)	43 (76.8%)	56 (100.0%)	​
Mean age - years (SD)	63.7 (11.9)	65.3 (12)	64.9 (11.9)	0.681
Performance status (ECOG)				
0/1	13 (100.0%)	40 (93.0%)	53 (94.6%)	0.328
2/3	0 (0.0%)	3 (7.0%)	3 (5.4%)	​
Surgical approach				
TH +/- BSO	10 (76.9%)	22 (51.2%)	32 (57.1%)	0.100
TH + BSO + LN +/- omentectomy	3 (23.1%)	21 (48.8%)	24 (42.9%)	​
Histology				
Endometrioid	9 (69.2%)	14 (32.6%)	23 (41.1%)	0.043
Serous	1 (7.7%)	19 (44.2%)	20 (35.7%)	​
Clear cell	0 (0.0%)	4 (9.3%)	4 (7.1%)	​
Carcinosarcoma	2 (15.4%)	2 (4.7%)	4 (7.1%)	​
Mixed	1 (7.7%)	4 (9.3%)	5 (8.9%)	​
Confined polyp or endometrium				
Polyp	3 (23.1%)	23 (53.5%)	26 (46.4%)	0.054
Endometrium	10 (76.9%)	20 (46.5%)	30 (53.6%)	​
Adjuvant treatment				
None	11 (84.6%)	25 (58.1%)	36 (64.3%)	0.196
Chemo +/- EBRT/BT	1 (7.7%)	5 (11.6%)	6 (10.7%)	​
EBRT and/or BT	1 (7.7%)	13 (30.2%)	14 (25.0%)	​
Progression				
No	13 (100.0%)	34 (79.1%)	47 (83.9%)	0.072
Yes	0 (0.0%)	9 (20.9%)	9 (16.1%)	​
Site progression				
Pelvic	0 (.%)	4 (44.4%)	4 (44.4%)	​
Abdominal	0 (.%)	3 (33.3%)	3 (33.3%)	​
Distant	0 (.%)	2 (22.2%)	2 (22.2%)	​
Death				
No	12 (92.3%)	37 (86.0%)	49 (87.5%)	0.550
Yes	1 (7.7%)	6 (14.0%)	7 (12.5%)	​

TH, total hysterectomy; BSO, bilateral salpingo-oophorectomy; LN, lymphadenectomy; SD, standard deviation, Chemo, chemotherapy; EBRT, external beam radiotherapy; BT, brachytherapy.

## Discussion

The prognosis and optimal management of FIGO 2023 IC EC tumors remain unsettled, with recent studies providing divergent insights. The importance of surgical staging in this scenario was highlighted by Hui et al. In their retrospective analysis of 40 patients with non-invasive USC, extrauterine disease was present in 45% of the cases. The authors concluded that surgical staging is an important step in the management of these patients [7]. Likewise, Boyraz et al. retrospectively analysed 33 patients with noninvasive USC and concluded that extrauterine spread at presentation is not uncommon (39% had already disease outside the uterus, especially omentum and adnexa), and comprehensive surgical staging, including omentectomy and pelvic and para-aortic lymph node dissection, is mandatory in this group of patients. Adjuvant treatment did not confer benefit [8]. These findings support the long-held view that USC should be regarded as a high-risk entity regardless of invasion, justifying comprehensive surgical staging with consideration of systemic therapy.

In contrast, Slaager et al recently published an observational cohort of pure SEIC and reported no recurrences or disease-related deaths, suggesting that lesions strictly confined to the endometrium may behave more indolently than previously assumed [9]. This challenges the traditional view, supported by earlier WHO classifications, that SEIC is uniformly aggressive with metastatic potential. By excluding superficially invasive disease, this study highlights the importance of rigorous pathological examination to avoid overtreatment.

Some studies also analysed the prognostic of histologies other than USC. Cuccinella et al demonstrated that among 97 patients with CS without myoinvasion, most had disease limited to the endometrium (56.7%), while 28.9% had tumor confined to a polyp and 14.4% had no residual disease. Twenty-nine patients (29.9%) experienced recurrence, mainly at distant sites. The 5-year recurrence-free survival was 63.5% and overall survival was 72.0%. Recurrence free and overall survival did not differ significantly according to tumor status (p = 0.99 and p = 0.43) or receipt of chemotherapy (p = 0.08 and 0.07) [10]. Regarding uterine CC, Sari et al showed that among 53 surgically staged non-invasive diseases, 12 (22.6%) were upstaged, including three (5.6%) with lymphatic spread. Most upstaged cases were stage IVB (9 patients), with the omentum being the most common metastatic site. After staging, 41 women (73.3%) had disease confined to the endometrium. Of these, 32% received no adjuvant therapy, while 68% did. The 5-year disease-free survival was higher in those receiving adjuvant treatment (100%) compared with observation (74.1%), although this difference did not reach statistical significance (p = 0.060) [11].

More recently, Jamienson et al showed that the prognostic impact is largely driven by p53 status rather than histotype alone. In a large multicenter study, stage IA p53abn endometrial cancers with no myometrial invasion demonstrated recurrence rates of 16%, with most relapses occurring at distant sites. Importantly, patients with residual endometrial disease had higher recurrence rates than those with tumors confined to a polyp. Adjuvant chemotherapy, with or without radiation, appeared to reduce recurrence risk, whereas brachytherapy alone offered little protection against distant relapse [12]. These findings suggest that molecular classification provides a more reliable framework for risk stratification than histotype or depth of invasion.

Our results are consistent with and extend these observations. We included only high-grade histologies to enrich tumors with p53 abnormality. Unfortunately, more than half of our patients were surgically treated with only total hysterectomy with or without bilateral salpingo-oophorectomy. Among 59 patients with noninvasive disease, three patients had already disease outside the uterus, highlighting the importance of surgical staging. Restricting the cohort to patients with FIGO stage IC, all nine recurrences occurred exclusively within the p53abn subgroup, while no patient with wild-type tumors experienced progression. Most recurrences in our cohort were outside the pelvis. The absence of recurrence in our wild-type cohort further supports the prognostic utility of molecular stratification, suggesting that surgical and systemic treatment intensity could be tailored to p53 status.

As a retrospective study, our analysis is limited by potential selection bias, small number of patients, heterogeneity in surgical staging, and non-standardized use of adjuvant therapy over time. Finally, the absence of uniform molecular testing beyond p53 status precluded a more comprehensive genomic classification, which may have provided additional prognostic refinement.

## Conclusion

Non myoivasive endometrial cancers with p53 abnormalities appear to have higher recurrence rates and poorer survival compared with wild-type tumors. In contrast, patients with wild-type disease demonstrated excellent outcomes, suggesting that overtreatment may be avoided in this subgroup. These findings should be interpreted with caution, given the retrospective design and require validation in larger, prospective studies before definitive treatment recommendations can be made. Future research should aim to better define the extent of surgical staging and to refine patient selection for adjuvant therapy, particularly chemotherapy.

## Data Availability

The original contributions presented in the study are included in the article/supplementary material, further inquiries can be directed to the corresponding author.
